# Characterization of the complete chloroplast genome sequence of *Impatiens pritzelii* (Balsaminaceae): an endemic species from China

**DOI:** 10.1080/23802359.2019.1689862

**Published:** 2019-11-18

**Authors:** Qian Wang, Wen-Qiao Li, Bo Ding, Hong-Ping Deng

**Affiliations:** a Chongqing Key Laboratory of Plant Resource Conservation and Germplasm Innovation, Institute of Resources Botany, School of Life Sciences, Southwest University, Chongqing, PR China;; b Biotechnology Research Center, Southwest University, Chongqing, PR China

**Keywords:** *Impatiens pritzelii*, chloroplast genome, phylogenomic analysis

## Abstract

This study was the first report about complete chloroplast genome of *Impatiens pritzelii* (Balsaminaceae). The plastome of *I. pritzelii* was found to possess a total length 152,487 bp with the typical quadripartite structure of angiosperms, containing two inverted repeats (IRs) of 25,684 bp separated by a large single-copy (LSC) region and a small single-copy (SSC) region of 83,290 and 17,829 bp, respectively. The plastid genome of *I. pritzelii* contains 113 genes, including 79 protein-coding genes, 4 ribosomal RNA genes, and 30 transfer RNA genes. The overall GC content of *I. pritzelii* plastid genome is 37.0% and the corresponding values in LSC, SSC, and IR regions are 34.9%, 29.8%, and 43.1%, respectively.

The genus *Impatiens* L. is a highly diversified genus with over 900 species distributed in highlands and mountains of the Old World, about 230 species (ca. 190 endemic species) distributed in China (Yuan et al. 2004; Yu et al. 2016). The species of *Impatiens pritzelii* J.D. Hooker is a Chinese endemic species distributed in NW of Hubei and E of Sichuan. A good knowledge of genomic information of this species would contribute to the formulation of protection strategy and the study of genome diversity and species diversity. In this study, we assembled and characterized its complete chloroplast genome (Gen-Bank accession number: MN 418389) from Illumina sequencing data.

Fresh leaves from one individual *I. pritzelii* were collected from Beibei District of Chongqing, China by Dr. Qian Wang. Meanwhile, the voucher specimen (Q. Wang JYS20190601) was collected and deposited in the herbarium of Southwest University (former herbarium of Southwest Normal University, SWCTU). The total genomic DNA was extracted using the modified CTAB method (Doyle and Doyle 1987). High quality total genomic DNA was extracted from ca.6 cm^2^ sections of the silica-dried leaf using improved Tiangen Plant Genomic DNA Kits, add the 4 μl RNAseA and 20 μl Proteinase K after incubated (65 °). The genome skimming sequencing was conducted, with 150 bp paired-end (PE) reads, on the Illumina HiSeq 2000 platform based the manufacturer’s protocol (Illumina, San Diego, CA, USA) by ORI-GENE, Beijing. Total 7.6 Gb data were generated. The raw reads were filtered for low-quality bases (PHRED <20) by NGSQC Toolkit version 2.3.3 (Patel and Jain 2012). The clean reads were then used to assemble the complete plastid genome using the plastid genomes *I. piufanensis* (NC_037401) as reference. We performed the assembly and annotation using Geneious version 10.1.3 (Biomatters Ltd., Auckland, New Zealand) and adjusted the genes manually to make sure that were maintained as open reading frames. IR boundaries for the draft plastome were confirmed by BLAST. Finally, we obtained a chloroplast genome of *I. pritzelii* and submitted the whole genome to GenBank (accession number: MN 418389).

The plastome of *I. pritzelii* was found to possess a total length 152,487 bp with the typical quadripartite structure of angiosperms, containing two inverted repeats (IRs) of 25,684 bp separated by a large single-copy (LSC) region and a small single-copy (SSC) region of 83,290 and 17,829 bp, respectively. The plastid genome of *I. pritzelii* contains 113 genes, including 79 protein-coding genes, 4 ribosomal RNA genes, and 30 transfer RNA genes. The overall GC content of *I. pritzelii* plastid genome is 37.0% and the corresponding values in LSC, SSC, and IR regions are 34.9%, 29.8%, and 43.1%, respectively.

We also used the coding sequences of *I. pritzelii* and another 12 plastomes to reconstruct a maximum likelihood tree through RAxML (Stamatakis 2014) under the GTRGAMMA substitution model, with 1000 bootstraps on CIPRES website (Miller et al. 2010). The result shows that *I. pritzelii*, *I. piufanensis,* and *Hydrocera triflora* formed the clade corresponding to Balsaminaceae (Figure 1).

**Figure 1.**
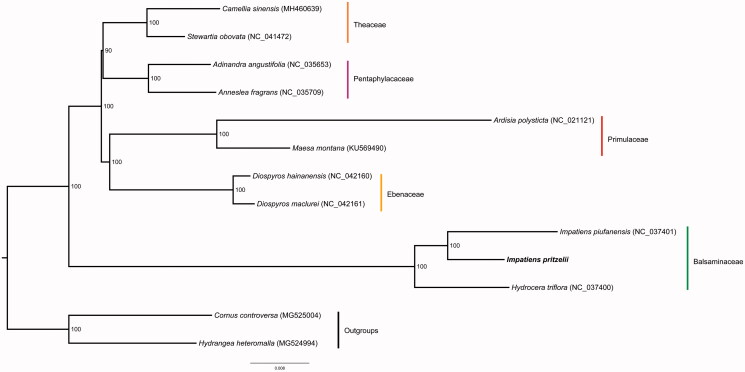
Phylogenetic tree based on the coding sequences of 13 plastomes. Bootstrap support value from 1000 replicates is shown above branches. All the plastome sequences are available in GenBank, with the accession numbers listed right to their scientific names. The new plastome obtained in this study is shown in bold font.
